# The effect of integrity of lesser tuberosity-medial calcar on postoperative outcome in the proximal humeral fracture

**DOI:** 10.1186/s13018-023-03851-0

**Published:** 2023-05-16

**Authors:** Hanru Ren, Lianghao Wu, Xu Zhang, Zhen Jian, Chengqing Yi

**Affiliations:** Department of Orthopaedics, Pudong Medical Center, Shanghai Pudong Hospital, Fudan University, No. 2800, Gongwei Road, Shanghai, 200120 China

**Keywords:** Proximal humerus fracture, Locking plate fixation, Medial calcar, Lesser tuberosity

## Abstract

**Background:**

In proximal humeral fractures, the medial calcar is often considered an important stabilizing structure. When the medial calcar is disrupted, some patients may have accompanying humeral lesser tuberosity comminution that has not been noticed. To investigate the impacts of comminuted fragments of lesser tuberosity and calcar on postoperative stability, CT results, number of fragments, cortical integrity, and the variation of neck-shaft angle were compared in patients with proximal humeral fractures.

**Materials and methods:**

From April 2016 to April 2021, this study included patients with senile proximal humeral fractures diagnosed by CT three-dimensional reconstruction with lesser tuberosity fractures and medial column injuries. The number of fragments in the lesser tuberosity and the continuity of medial calcar were evaluated. Postoperative stability and shoulder function were evaluated by comparing changes in neck-shaft angle and the DASH upper extremity function score from 1 week to 1 year after the operation.

**Results:**

A total of 131 patients were included in the study, and the results showed that the number of fragments of the lesser tuberosity was related to the integrity of the medial cortex of the humerus. That is, when there were more than two lesser tuberosity fragments, the integrity of humeral medial calcar was poor. The positive rate of the lift-off test was higher in patients with lesser tuberosity comminutions 1 year after surgery. In addition, patients with more than two lesser tuberosity fragments and continuous destruction of the medial calcar had large variations in the neck-shaft angle, high DASH scores, poor postoperative stability, and poor recovery of shoulder joint function 1 year postoperatively.

**Conclusion:**

The number of humeral lesser tuberosity fragments and the integrity of the medial calcar were associated with the collapse of the humeral head and the decrease in shoulder joint stability after the proximal humeral fracture surgery. When the number of lesser tuberosity fragments was greater than two and the medial calcar was damaged, the proximal humeral fracture had poor postoperative stability and poor functional recovery of the shoulder joint, which required auxiliary internal fixation treatment.

## Introduction

Proximal humeral fracture is a common senile osteoporotic fracture, mostly occurring in elderly women [1–3]. With the advent of an aging society, the number of patients with proximal humeral fractures is increasing year by year. The open reduction and internal fixation (ORIF) with a lateral locking plating (LLP) is currently the most commonly used surgical method. However, studies have reported that at least 50% of proximal humeral fracture patients have obvious displacements that affect the postoperative shoulder joint function [4, 5].

The medial cortex of the proximal humerus, the medial calcar, is an important stabilizing structure. Studies have concluded that the integrity of the medial structure is closely related to postoperative complications. If the medial cortex cannot provide effective support during the operation, the medial column collapse will occur even with the fixation of the calcar screws [6, 7]. The collapsed humeral head is associated with long-term postoperative pain and functional limitations of the shoulder joint. Therefore, maximizing the restoration of the integrity of the humeral medial calcar is vital for proximal humeral fracture surgery. The lesser humeral tuberosity is located at the anteromedial side of the proximal humerus and is also the attachment point of the subscapular muscle [8]. Free lesser tuberosity fragments are often seen in 3-part and 4-part fractures. However, the anteromedial free lesser tuberosity fragments are usually not fixed, and few studies have analyzed the impact of the lesser tuberosity fragments on proximal humerus fracture.

The medial margin of the lesser tuberosity is connected to the medial calcar. Through 3D-CT reconstruction, it was found that lesser tuberosity comminutions were often accompanied by more severe medial calcar fractures. Researchers have now begun to pay attention to the impingement of lesser tuberosity fractures on proximal humerus fracture treatments. By biomechanical research, Katthagen et al. concluded that fixing lesser tuberosity helped improve the stability of proximal humerus fractures [9]. However, studies on this topic are still scarce. There is no clinical study evaluating the influence of the lesser tuberosity fractures and their associated medial calcar fractures on the postoperative stability of proximal humerus fractures.

In this study, CT results, the number of fragments, the integrity of medial calcar, the variation of neck-shaft angle, and the shoulder function scores of patients were compared to investigate the impacts of comminuted fragments of lesser tuberosity and medial calcar on postoperative stability. Our preliminary hypothesis was that comminuted fracture of the lesser tuberosity predicts the occurrence of destruction of medial calcar integrity. In addition, fragmentation of the lesser tuberosity and injury of the medial calcar will affect the function of the shoulder joint after surgery.

## Materials and methods

From April 2016 to April 2021, this study retrospectively analyzed elderly patients with proximal humerus fractures accompanied by lesser tuberosity fractures and medial cortical injuries, which were diagnosed by 3D-CT reconstruction. The following patients were included according to the inclusion criteria: patients above 60 years of age with 3-part or 4-part proximal humeral fractures with lesser tuberosity fragments and medial cortical injuries; patients who were followed up for more than 1 year and treated with LLP in the proximal humerus. Exclusion criteria: patients admitted to the hospital more than 5 days after injury; patients with other multiple fractures or multiple injuries; patients with other severe shoulder joint diseases, shoulder motion, and sensory deficits before the injury; patients with auxiliary steel cables, or any auxiliary plate fixation; and patients who had obvious postoperative displacements of the calcar screws (as the wrong placement position of the calcar screw may lead to the collapse of the humeral head after surgery, resulting in the displacement of the fractures).

All patients with proximal humeral fracture confirmed by emergency X-ray were then diagnosed by 3D-CT reconstruction to determine the type and the extent of fractures. The patients were scheduled for surgery within 5 days of hospitalization and the surgeons involved were all orthopedic physicians from our hospital. All surgeries were performed by a deputy chief or chief physician. The surgical Approach was delto-pectoral approach. Moreover, all patients underwent open reduction and internal fixation surgery, utilizing proximal humeral locking plates (PHILP) for fixation. No additional fixation of the free lesser tuberosity fragment was performed intraoperatively. A standardized rehabilitation exercise process was adopted after the operation. The affected limb was immobilized in suspension for 3 weeks. Passive shoulder joint function exercise was carried out from the 3rd week to the 9th week. After 9 weeks, the active non-weight-bearing shoulder joint activity was gradually increased. After 12 weeks, the shoulder joint begins to perform weight-bearing exercises.

**Fig. 1:**
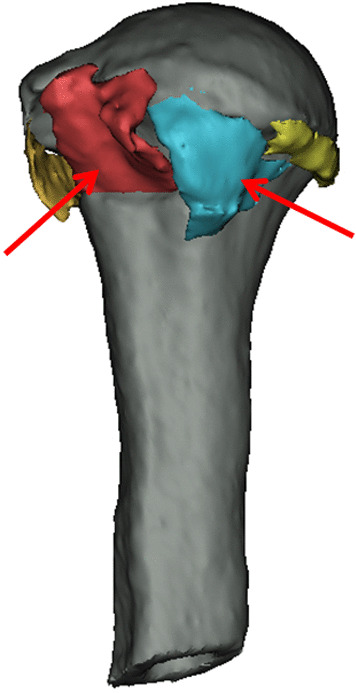
The red arrow shows lesser tuberosity fragments

In this study, all patients were diagnosed by two senior attending physicians in the trauma department through X-ray and 3D-CT reconstruction prior to the operation. The fracture classification, the number of bone fragments of the lesser tuberosity, and the continuity of medial calcar were evaluated and the results were recorded. The judgment of the number of fragments of lesser tuberosity is shown in Fig. 1.

**Fig. 2:**
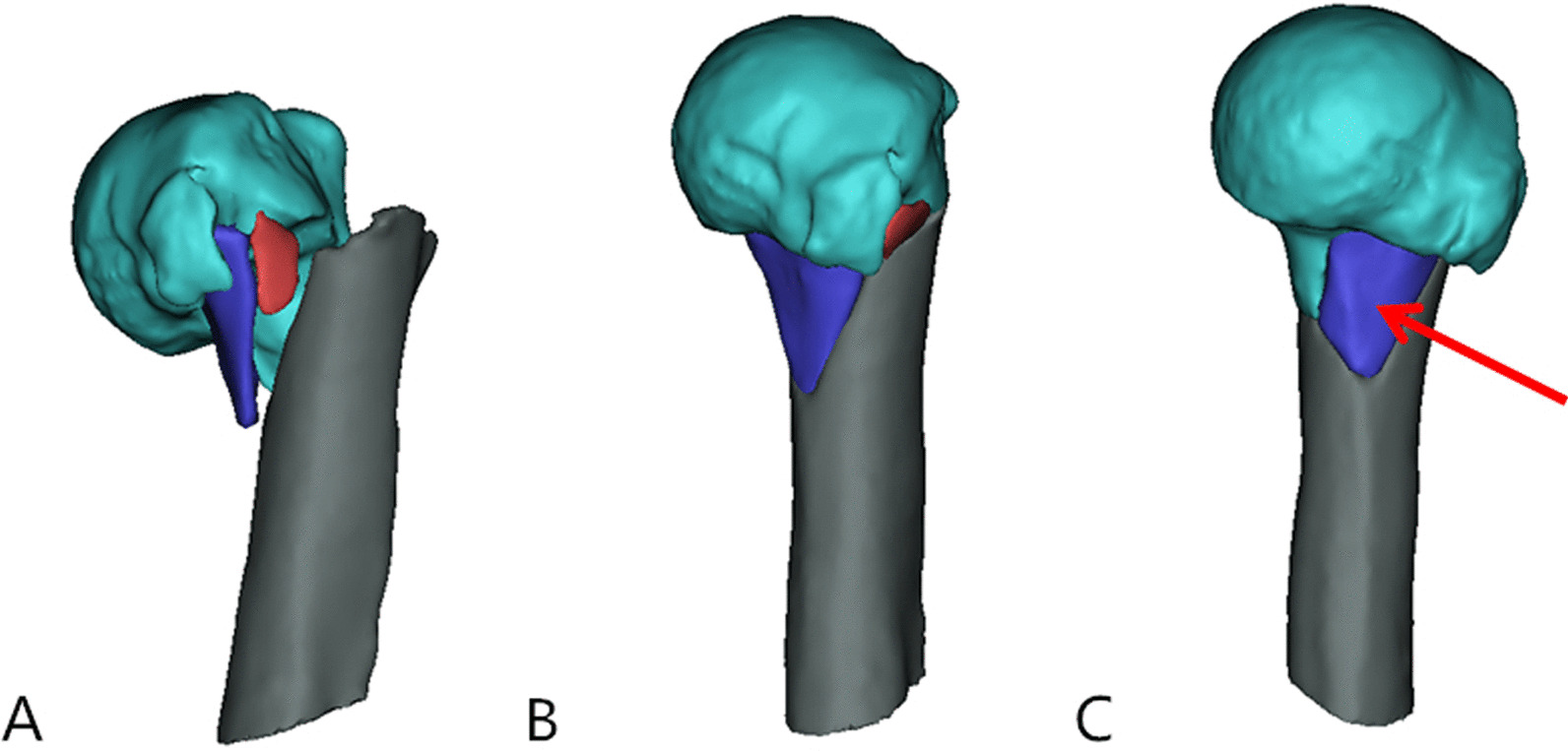
Observe the continuity of the patient's medial calcar. **A** Preoperative 3D-CT reconstruction view. **B** Preoperative CT data were used for virtually reduced fractures. **C** After virtual reduction, observe whether there are disruptive bone defects in the medial side of the patient (The red arrow shows medial calcar fragment)

Judgment of continuity of the medial calcar: virtual reduction based on 3D-CT reconstruction results was performed for all patients before the operation. If the patient had significant bone defects and discontinuity in the medial cortex after reduction, it suggested that the medial calcar was disrupted in this patient, and the reason for which was usually the fragmentation of the cortex (Fig. 2).

Stability: the postoperative stability of the patient was determined by the degree of collapse of the humeral head, which was assessed by the variation of the neck-shaft angle observed in the radiograph of the anteroposterior shoulder joint. The change of the neck-shaft angle was measured immediately after the operation and 1 year after the operation, respectively. The varus angulation change of neck-shaft angle > 20° indicated that the patient experienced collapsed humeral head [10–12].

Lift-off test: the insertion point of the subscapular muscle was located at the lateral edge of the lesser tuberosity. Considering the postoperative injury and repair of the subscapular muscle, a lift-off test was performed 12 months after the operation. Evaluation method: the palm of the affected limb of the patient was placed on the lower back and the patient was asked to rotate the arm inward to lift the hand off the back. The test was considered positive if the patient was unable to lift the arm off the back or to lift by extending the elbow or shoulder [13].

All statistical analyses were performed using SPSS 22.0 statistical software (SSPS, Chicago, IL). Measurement data are presented as means and standard deviations. Data were analyzed using one-way analysis of variance (ANOVA), and intergroup comparisons were conducted using the Student–Newman–Keuls method. In all tests, a *P* value less than 0.05 was considered statistically significant.

## Results

A total of 412 patients with proximal humeral fractures participated in the study. Among them, 175 senile patients were diagnosed with lesser tuberosity fragments and medial cortex injuries by CT, including 6 with multiple traumas, 3 with partial loss of motility of upper limbs due to pre-injury cerebral infarction, 12 with lost follow-ups, 14 preoperative chronic shoulder disease, and 9 with postoperative radiographs indicating the wrong placement of humeral medial calcar screws. Finally, 131 patients were eligible for the study. The causes of injuries included 102 falls at home and 29 traffic accidents. The age, sex, weight, length of stay, and follow-up time of the patients are shown in Table 1. The mean age was 71.6 ± 7.13 years and the average follow-up time was 15.33 ± 3.17 months.

**Table1:** Demographic data and baseline characteristics

	Value
Age (years)	71.6 ± 7.13
Gender (male/female)	49/82
Weight (kg)	67.01 ± 4.88 (52–82)
Length of stay (day)	9.12 ± 2.03 (5–12)
follow-up (month)	15.33 ± 3.17

Data are presented as mean ± SD (range) or No. of patients

Overall 131 patients with proximal humeral fractures were identified, there were 51 cases with 1 lesser tuberosity fragment, 23 with 2 lesser tuberosity fragments, and 57 with more than two fragments. 88 cases had integral medial calcar and 43 cases had discontinuous calcar. It was found that when the calcar was disrupted, the fragmentation of the lesser tuberosity was exacerbated. Besides, patients with undamaged calcar had relatively intact lesser tuberosity.

**Fig. 3:**
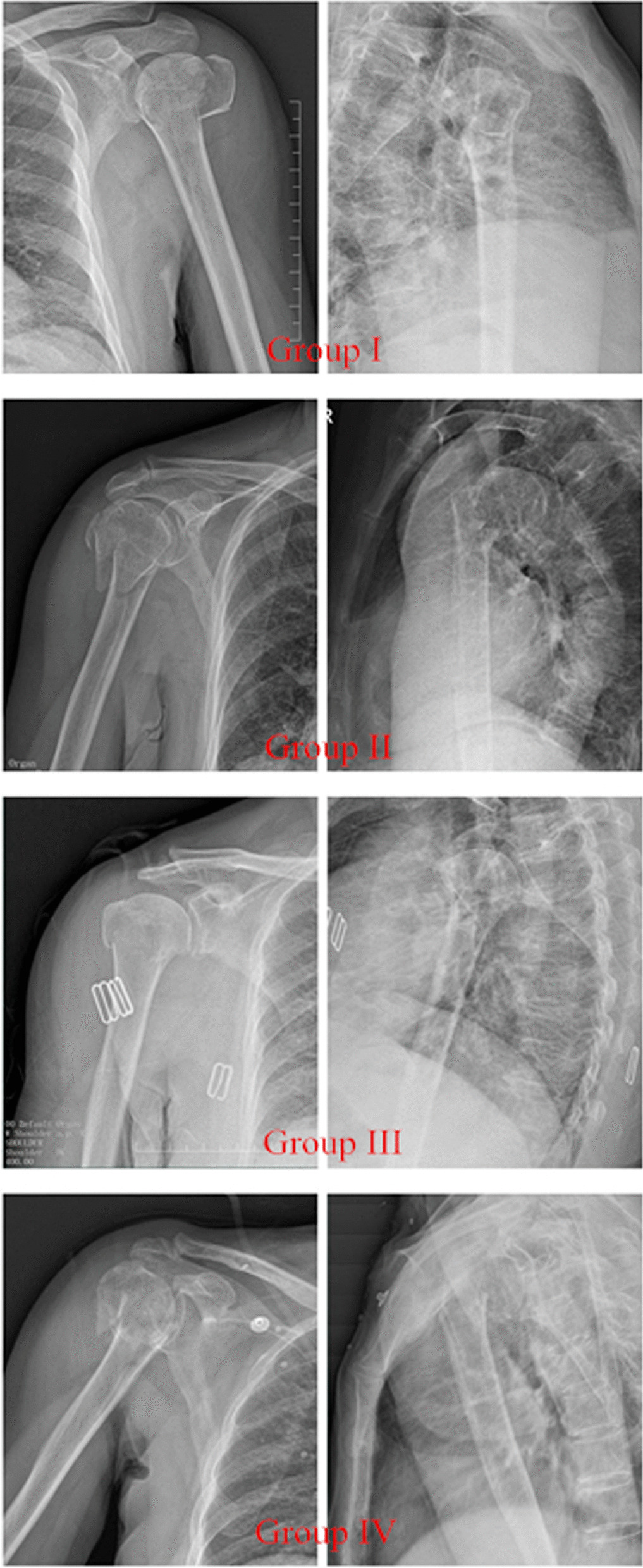
The A-P view and axillary view in Group I–IV

Patients were divided into four groups (Fig. 3). Group I: patients with ≤ 2 lesser tuberosity fragments and an intact calcar. Group II: patients with ≤ 2 lesser tuberosity fragments and a disrupted calcar. Group III: patients with > 2 lesser tuberosity fragments and an intact calcar. Group IV: patients with > 2 lesser tuberosity fragments and a disrupted calcar. The specific number of cases is shown in Table 2.

**Table 2:** The characteristics of antero-medial fragment for proximal humerus fracture patients

Variable	Number (%)
Humeral calcar intact/injury	88/43
Lesser tubercle fragment quantity = 1	51/138
Lesser tubercle fragment quantity = 2	23/138
Lesser tubercle fragment quantity > 2	57/138

**Fig. 4:**
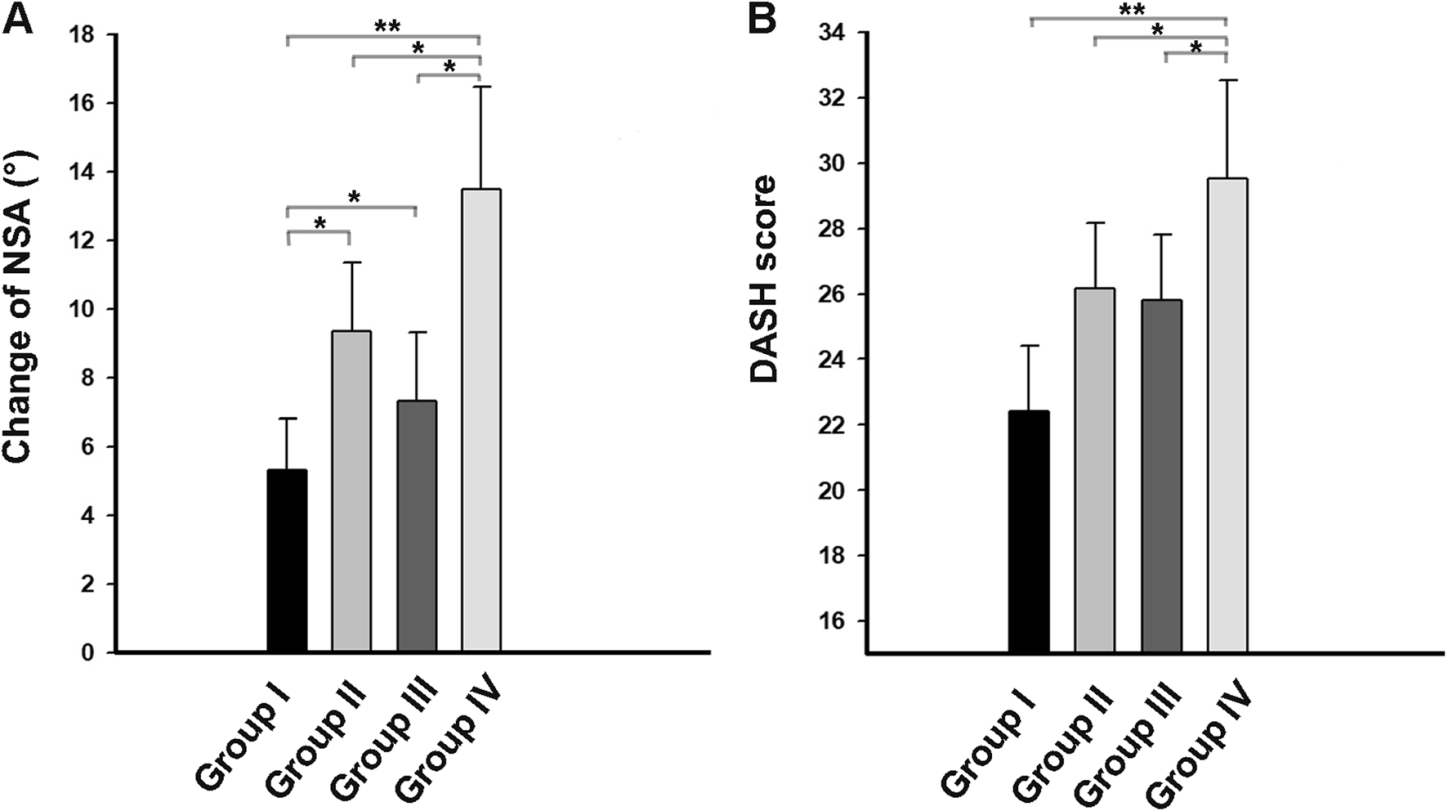
**A** The change of neck–shaft angle by different groups. **B** The upper limb DASH scores by different groups (*P < 0.01, **P < 0.005)

The changes in the neck-shaft angle in different groups were measured, and the difference between the groups was significant. The variations of the neck-shaft angle in Group IV were the greatest 1 year after the operation, at 13.48° ± 8.91°. The results showed significant differences between Group IV and the other three groups. In addition, Group II and Group III also indicated statistical differences from Group I. And there was no difference between Group II and Group III. On the other hand, results suggested that a total of 12 patients had neck-shaft angle changes of more than 20° 1 year after the operation accompanied by the collapse of the humeral head. Notably, all 12 patients belonged to Group IV. Details are described in Table 3 and Fig. 4.

**Table 3:** Comparison of the postoperative and the final follow‑up patients characteristics between groups

Description	Group				
	I (*n* = 70)	II (*n* = 15)	III (*n* = 18)	IV (*n* = 28)	*P* value
Change of NSA (°)	5.32 ± 2.91°	9.37 ± 3.53°	7.33 ± 3.71°	13.48 ± 8.91°	< 0.001*
DASH score	22.41 ± 3.24	26.15 ± 3.16	25.81 ± 4.97	29.53 ± 7.04	0.011*
VAS score	1.74 ± 0.81	1.71 ± 0.90	1.51 ± 0.84	1.91 ± 1.37	0.073
Lift-off test (±)	22/48	6/11	15/3	26/2	–

*NSA* neck shaft angle, *DASH* disabilities of the arm, shoulder and hand

^*^P < 0.05 was considered significant

The upper limb DASH scores and VAS scores of patients in different groups are shown in Table 3 and Fig. 3. The upper limb DASH scores in Group IV were significantly higher than the other three groups. The results indicated that the postoperative function of patients in Group IV was poor. However, there was no significant difference in VAS scores between groups 12 months at 12 months postoperatively. Additionally, according to the result of the lift-off test, the positive rate of patients in Group III and Group IV was significantly higher than those in Group I and Group II, which was related to the injury of the subscapular muscle.

## Discussion

Proximal humeral fracture is a common osteoporotic fracture in the elderly. It usually occurs in low-energy fragility fractures. With the advent of global aging, the incidence of proximal humeral fracture is increasing every year [14, 15]. However, the treatment of proximal humeral fracture with the comminuted medial wall remains controversial because of the high incidence of postoperative complications [1, 16]. The integrity of the medial wall of the proximal humerus plays an important role in its postoperative stability and its biomechanical properties are similar to those of the proximal femur [12, 17]. At present, it has been accepted that comminuted fracture of the medial calcar with the bone defect may result in the varus of the humeral head, which is the most common pattern of internal fixation failure [18]. ORIF using PHILOS plate (Synthes) is still the most commonly used surgical method for the treatment of proximal humeral fractures [19]. To prevent varus deviation of the humeral head, the medial calcar screws must be inserted along the inferior tangential direction of the medial cortex to enhance fracture stability during the treatment of a injured medial calcar. However, in some patients, the medial cortex is severely disrupted and the calcar continuity cannot be maintained after reduction; or in the case of extremely serious osteoporosis, varus deformity of the humeral head may occur even after fixation with the calcar screws. In view of the high incidence of postoperative complications, some investigators have suggested that an adjuvant plate or fibular strutallograft (FSA) could be used for additional fixation in addition to PHILOS [20, 21]. The plates can be placed in various positions, including the anterior and medial to the proximal humerus [6, 22]. However, the technique of anterior plate assisted internal fixation has rarely been described, and few studies have been performed on the effect of lesser tuberosity fractures and their associated medial calcar disruption on patients with proximal humerus fractures.

In recent studies, researchers have begun to pay attention to the role of the lesser tuberosity in the stability of the proximal humerus. Katthagen JC et al. conducted a biomechanical study on whether the 4-part proximal humeral fractures were stabled by the lesser tuberosity fixation. The results have shown that the use of screw-assisted fixation in the 4-part proximal humeral fractures was effective in improving the stability of the proximal humerus [9]. This study also demonstrated the key of the lesser tuberosity on the structural stability of the proximal humerus. In our study, a correlation was found between the comminution of the lesser tuberosity (fragments > 2) and the destruction of medial calcar. The number of patients in Group I and Group IV was 98, which was significantly higher than in Group II and Group III. Considering that the medial aspect of the lesser tuberosity is connected with the humeral medial calcar, we believe that if the lesser tuberosity is comminuted and displaced on X-ray, it indicates the destruction of the calcar. In addition, when the calcar disruption, the change of the neck-shaft angle was 9.37° ± 3.53° for an intact lesser tuberosity and 13.48° ± 8.91° for a comminuted lesser tuberosity. Therefore, the fragmentation of the lesser tuberosity further predicts the occurrence of postoperative humeral head collapse, which may be related to the following reasons. When there is lesser tuberosity comminution and humeral medial calcar injury, the patient’s fracture is severely displaced, and the anterior varus of the humeral head often occurs. At this point, the reduction becomes more difficult and there is a lack of support for the medial side of the humerus after the operation. However, the free tuberosity fragment was not fixed intraoperatively and the proximal humerus was unsupported anteriorly. Besides, patients with preoperative osteoporosis have relatively slow postoperative functional recovery, which further aggravates the osteoporosis, and may lead to inadequate s postoperative screw support.

In our study, although there was no difference in VAS scores between groups 1 year after the operation, the results of DASH upper limb function scores were similar to the variations of humeral neck-shaft angle. It was believed that this was due to the functional impact of the humeral head collapse. Furthermore, regarding the fact that the patient's lesser tuberosity comminution may lead to the injury of subscapular muscle, the positive rate of lift-off test 1 year after surgery was analyzed. As expected, the positive rate of lift-off test was higher in Group III and Group IV patients 1 year after the operation, indicating that lesser tuberosity comminution may affect the function of the subscapularis muscle, which needs further research and analysis. If the function of the subscapularis muscle is affected, it will not only lead to the loss of shoulder adduction function but also further lifting and pronation function. The unsatisfactory reduction of the fracture at the lesser tuberosity may affect the matching of the humeral head and the shoulder glenoid cavity, resulting in incorrect trajectory of the humeral head over the shoulder glenoid cavity (similar to the off-track of Hill-Sachs injury) [23], and even the possibility of postoperative shoulder instability. Therefore, it is not sufficient to rely on the LLP solely to treat medial calcar disruption accompanied by lesser tuberosity comminution. The medial bone grafting combined with steel cable can be used to bind and fix the fractures in some patients. However, it may not able to completely tie the severely fragmented anterior lesser tuberosity fragments, and the anterior plate is required to assist in fixation.

The study has some limitations. First, it is a retrospective study in which some patients with relatively severe fractures of the lesser tuberosity opted for conservative treatment, while other patients with severe fractures of the lesser tuberosity and medial calcar were treated with shoulder arthroplasty, which may have altered the proportion of patients in the different groups. In addition, the range of motion of the shoulder joint was not evaluated in all patients. Future prospective investigations should clarify the results of this study.

## Conclusion

More than two humeral lesser tuberosity fragments and medial calcar disruption result in significantly increased postoperative changes in the neck-shaft angle, lower postoperative stability, and higher DASH functional scores 1 year after surgery. In addition, patients with comminution of the lesser tuberosity had a higher lift-off test rate 1 year after surgery, indicating that the subscapularis muscle is difficult to repair in these patients. Therefore, after the diagnosis of comminuted fracture of the lesser tuberosity and medial calcaneal injury, a medial bone graft combined with an auxiliary cable or anterior-assisted internal fixation is recommended to reduce the incidence of postoperative complications.

## Data Availability

The datasets used and analyzed during the current study are available from the corresponding author on reasonable request.

## References

[1] Mease SJ, Kraeutler MJ, Gonzales-Luna DC, Gregory JM, Gardner MJ, Choo AM (2021). Current controversies in the treatment of geriatric proximal humeral fractures. J Bone Joint Surg Am.

[2] Boyer P, Couffignal C, Bahman M, Mylle G, Rousseau MA, Dukan R (2021). Displaced three and four part proximal humeral fractures: prospective controlled randomized open-label two-arm study comparing intramedullary nailing and locking plate. Int Orthop.

[3] Yahuaca BI, Simon P, Christmas KN, Patel S, Gorman RA, Mighell MA (2020). Acute surgical management of proximal humerus fractures: ORIF vs. hemiarthroplasty vs. reverse shoulder arthroplasty. J Shoulder Elbow Surg.

[4] Favorito P, Kohrs B, Donnelly D (2021). Proximal humeral fractures treated with an intramedullary cage and plate: clinical and radiographic outcomes at a minimum of 1 year postoperatively. J Shoulder Elbow Surg.

[5] Maier D, Jaeger M, Izadpanah K, Strohm PC, Suedkamp NP (2014). Proximal humeral fracture treatment in adults. J Bone Joint Surg Am.

[6] Zhang Y, Wan L, Zhang L, Yan C, Wang G (2021). Reduction and fixation of proximal humeral fracture with severe medial instability using a small locking plate. BMC Surg.

[7] Mehta S, Chin M, Sanville J, Namdari S, Hast MW (2018). Calcar screw position in proximal humerus fracture fixation: Don't miss high!. Injury.

[8] Corona K, Cerciello S, Ciolli G, Proietti L, D'Ambrosi R, Braile A, Toro G, Romano AM, Ascione F (2021). Clinical outcomes and joint stability after lateralized reverse total shoulder arthroplasty with and without subscapularis repair: a meta-analysis. J Clin Med.

[9] Katthagen JC, Michel P, Raschke MJ, Sussiek J, Frank A, Wermers J (2021). The forgotten fragment: additional lesser tuberosity fixation of 4-part proximal humeral fractures-a biomechanical investigation. J Shoulder Elbow Surg.

[10] Bouliane M, Silveira A, AlEidan A, Heinrichs L, Kang SH, Sheps DM (2020). Factors associated with maintaining reduction following locking plate fixation of proximal humerus fractures: a population-based retrospective cohort study. JSES Int.

[11] Oppeboen S, Wikeroy AKB, Fuglesang HFS, Dolatowski FC, Randsborg PH (2018). Calcar screws and adequate reduction reduced the risk of fixation failure in proximal humeral fractures treated with a locking plate: 190 patients followed for a mean of 3 years. J Orthop Surg Res.

[12] Lin SJ, Tsai YH, Yang TY, Shen SH, Huang KC, Lee MS (2015). Medial calcar support and radiographic outcomes of plate fixation for proximal humeral fractures. Biomed Res Int.

[13] Ladermann A, Collin P, Zbinden O, Meynard T, Saffarini M, Chiu JC (2021). Diagnostic accuracy of clinical tests for subscapularis tears: a systematic review and meta-analysis. Orthop J Sports Med.

[14] Howard L, Berdusco R, Momoli F, Pollock J, Liew A, Papp S (2018). Open reduction internal fixation vs non-operative management in proximal humerus fractures: a prospective, randomized controlled trial protocol. BMC Musculoskelet Disord.

[15] Aaron D, Shatsky J, Paredes JC, Jiang C, Parsons BO, Flatow EL (2012). Proximal humeral fractures: internal fixation. J Bone Joint Surg Am.

[16] Erdogan M, Desteli EE, Imren Y, Uzturk A, Kilic M, Sezgin H (2014). The effect of inferomedial screw on postoperative shoulder function and mechanical alignment in proximal humerus fractures. Eur J Orthop Surg Traumatol.

[17] Ott N, Hackl M, Prescher A, Scaal M, Lanzerath F, Muller LP (2022). The effect of long calcar screws on the primary stability of 3-part, varus impacted proximal humeral fractures compared to short calcar screws: a real fracture simulation study. Arch Orthop Trauma Surg.

[18] Ponce BA, Thompson KJ, Raghava P, Eberhardt AW, Tate JP, Volgas DA (2013). The role of medial comminution and calcar restoration in varus collapse of proximal humeral fractures treated with locking plates. J Bone Joint Surg Am.

[19] Zhao L, Qi YM, Yang L, Wang GR, Zheng SN, Wang Q (2019). Comparison of the effects of proximal humeral internal locking system (PHILOS) alone and PHILOS combined with fibular allograft in the treatment of neer three- or four-part proximal humerus fractures in the elderly. Orthop Surg.

[20] Sun Q, Wu X, Wang L, Cai M (2020). The plate fixation strategy of complex proximal humeral fractures. Int Orthop.

[21] Little MT, Berkes MB, Schottel PC, Lazaro LE, LaMont LE, Pardee NC (2014). The impact of preoperative coronal plane deformity on proximal humerus fixation with endosteal augmentation. J Orthop Trauma.

[22] Park SG, Ko YJ (2019). Medial buttress plating for humerus fractures with unstable medial column. J Orthop Trauma.

[23] Itoi E (2017). 'On-track' and 'off-track' shoulder lesions. EFORT Open Rev.

